# Comparative Evaluation of the Volatile Profile of the Essential Oil, the Hydrolate and the Plant Material from *Origanum vulgare* subsp. *virens* Grown in Portugal

**DOI:** 10.3390/foods14244175

**Published:** 2025-12-05

**Authors:** Carolina Salles Freire, Maria das Graças Cardoso, Orlanda Póvoa, Noémia Farinha, David Lee Nelson, Alexandra M. Machado, Ana Cristina Figueiredo

**Affiliations:** 1 Chemistry Department, Federal University of Lavras (UFLA), Lavras 37203-202, Minas Gerais, Brazil; carolina.freire2@estudante.ufla.br (C.S.F.); mcardoso@ufla.br (M.d.G.C.); 2 VALORIZA—Centro de Investigação para a Valorização de Recursos Endógenos, Instituto Politécnico de Portalegre, Praça do Município 11, 7300-110 Portalegre, Portugal; opovoa@ipportalegre.pt (O.P.); nfarinha@ipportalegre.pt (N.F.); 3 Postgraduate Program in Biofuels, Federal University of the Jequitinhonha and Mucuri Valleys, Diamantina 39803-371, Minas Gerais, Brazil; dleenelson@gmail.com; 4 Centre for Ecology, Evolution and Environmental Changes (CE3C) & Global Change and Sustainability Institute (CHANGE), Faculdade de Ciências da Universidade de Lisboa, Biotecnologia Vegetal, DBio, Campo Grande, 1749-016 Lisboa, Portugal; ampmachado@fc.ul.pt

**Keywords:** oregano, chemotypes, phytochemistry, Lamiaceae, extraction procedures, chemical descriptors

## Abstract

Oregano (*Origanum vulgare* L.) is one of the most important aromatic plants in the world, recognized for its applications in food and traditional medicine, as well as for its biological potential and chemical diversity. This study investigated the chemical diversity of 12 accessions of *Origanum vulgare* subsp. *virens* from Portugal, grown in the wild and on an experimental field (EF), through a comparative analysis of their essential oils, hydrolate volatiles, and the volatiles obtained by headspace solid-phase microextraction (HS-SPME). The compounds were identified by gas chromatography coupled with mass spectrometry and quantified by gas chromatography with a flame ionization detector. The yields of the essential oil ranged from 1.5 to 3.3% (*v*/*w*). A total of 70 compounds were identified in the essential oil, 74 compounds in the hydrolate volatiles, and 48 compounds by HS-SPME. Cluster analysis separated the 36 samples into two main groups, corresponding to the linalool (35–77%) and the terpene-phenolic chemotypes [thymol (24–82%) and carvacrol (18–93%)]. PCA clearly separated the three methodologies of volatiles extraction while keeping similar chemotypes. With few exceptions, the wild and the corresponding EF-grown plants provided comparable volatile profiles. The choice of analytical method can influence the chemical profile, which demonstrates the need for a more comprehensive approach to understanding the chemical descriptors from Portuguese oregano.

## 1. Introduction

*Origanum vulgare* L., commonly known as oregano, belongs to the Lamiaceae family and is one of the most widely consumed and commercialized medicinal and aromatic plants in the world. *O. vulgare* is native from and widely distributed in the Mediterranean region and is recognized for its high variability, which includes many subspecies and hybrids [1]. According to studies by Ietswaart [2], 38 species, 6 subspecies, and 17 hybrids were recognized within the *Origanum* genus. In Portugal, two subspecies exist (*O. vulgare* subsp. *vulgare* L. and *O. vulgare* subsp. *virens* (Hoffmanns. & Link) Bonnier & Layens), the most common of which is *O. vulgare* subsp. *virens* [3].

Oregano has been used since ancient times in cooking, as a seasoning, and in folk medicine to treat a variety of ailments including digestive and respiratory disorders, as well as skin infections [4,5]. Its economic and pharmacological importance is due to its rich composition of secondary metabolites, which highlights the compounds present in its essential oils (EOs). These EOs are responsible not only for its characteristic aroma, but also for a wide range of biological activities, such as antimicrobial, antioxidant, anti-inflammatory, antiparasitic, antitumor, and wound healing ability [6,7,8,9,10,11].

*O. vulgare* is recognized for its great variability, both morphological and chemical [12]. The chemical composition of its essential oil is complex and can vary significantly, which leads to the classification of different chemotypes. According to Figueiredo [13], chemotypes are distinct chemical groups within the same plant species that, although phenotypically similar, differ in the composition or proportion of their chemical constituents.

Zinno et al. [1] described one of the principal factors leading to this variability as the plant’s genetic background. In addition, physiological and related factors (organ development, pollinator activity cycle, type of plant material and secretory structure, seasonal variation, mechanical and chemical injuries) as well as environmental conditions (climate, pollution, diseases and pests and edaphic factors) significantly contribute not only to differences in volatiles yield, but also to variations in the relative amounts of their main components [14].

The essential oil from *O. vulgare* is characterized by the presence of monoterpene and sesquiterpene hydrocarbons, as well as phenol-like oxygen-containing monoterpenes [15]. The constituents that frequently dominate the chemical profile include the isomers thymol and carvacrol, myrcene, *p*-cymene, sabinene, γ-terpinene, linalool, terpinen-4-ol, α-terpineol, and linalyl acetate [3,4,15].

A wide variety of studies have evaluated the diversity of oregano’s composition, but most of these studies focus solely on the essential oil obtained by hydrodistillation or steam distillation. Although distillation is one of the main methods for large-scale extraction, it can induce the formation of artifact compounds. The prolonged heating process can cause the degradation of thermally sensitive compounds, hydrolysis reactions, or molecular rearrangements. It is important to mention that some of these artifacts may be even important from the industrial point of view (chamazulene for instance), and that whatever the methodology of extraction, they all have pros and cons, and none will entirely reflect the plant’s natural composition.

Hydrolates are distilled aromatic waters that result as a co-product of hydro- or steam distillation [16]. Many times considered a by-product, and often discarded, hydrolates are colloidal suspensions of essential oil droplets, also rich in other water-soluble compounds. Considered milder than the corresponding essential oils, they can have various food, beverages, cosmetic, pharmaceutical, and agroforestry applications [16]. Recent studies have shown that *O. vulgare* hydrolates exhibit considerable chemical diversity, with oxygen-containing monoterpenes (such as linalool, thymol, and carvacrol) dominating the composition, followed by monoterpene hydrocarbons (such as γ-terpinene), although in different proportions compared to essential oils. This variability reflects the plant’s chemotype and is influenced by genetic, environmental, and extraction-related factors [17,18].

The headspace solid-phase microextraction (HS-SPME) technique, while not producing an EO, allows one to determine the chemical profile of the volatile components naturally emitted by the plant, which are ecologically relevant and have potential applications in biological assays (e.g., fumigation tests against fungi), pest management, and aroma-based product development [19].

Because of its value in different sectors, both oregano herb and essential oil are prone to economically motivated adulteration (EMA) [20,21] and chromatographic techniques, along with other methodologies (macroscopic, microscopic, chemical and/or molecular), can contribute to detect fraudulent adulteration of herbs, essential oils, and volatiles. Therefore, a multifaceted approach to chemical composition, taking into account the different methods of obtaining volatile compounds, is essential for a more robust assessment of the chemical variability of *O. vulgare* volatile constituents. This study sought to investigate the chemical diversity of different accessions of *Origanum vulgare* subsp. *virens* from South Portugal through a study that compares the volatile profiles obtained from wild and cultivated plants by three different methods: (1) the essential oil isolated by hydrodistillation, (2) the volatiles obtained from the hydrolate, by liquid/liquid extraction, and (3) the headspace volatiles obtained by HS-SPME.

## 2. Materials and Methods

### 2.1. Plant Material

The samples of *Origanum vulgare* subsp. *virens* analyzed in this study were obtained from six wild accessions (S) collected from different locations (Table 1), in the Alentejo region in Portugal, or from the same cultivated accessions (C) in the Experimental Field of the Escola Superior de Biociências de Elvas/Instituto Politécnico de Portalegre (ESBE/IPP), totaling 12 oregano samples (Table 1). Plants were previously morphologically characterized supported on the 2011 ECPGR Working Group on Medicinal and Aromatic Plants Draft Descriptor List for *Origanum vulgare* [22]. These morphological descriptors are publicly available from Portuguese Plant Germplasm Bank (BPGV) in GrinGlobal database platform [23]. The oregano accessions were harvested during flowering, at the end of May 2024, and air-dried in the shade at room temperature.

### 2.2. Extraction of the Essential Oil

Oregano essential oils were isolated by hydrodistillation for 3 h using a Clevenger apparatus according to the European Pharmacopoeia [24]. On average, ≈ 200 g of dry plant material were used in each distillation. The distillation rate was 3 mL/min, and the EO and hydrolate samples were stored at −20 °C, until analysis.

### 2.3. Extraction of Volatile Compounds from the Hydrolate

The volatile compounds from the hydrolate were obtained by liquid–liquid extraction with laboratory-distilled pentane as the solvent, using a ratio of three volumes of pentane per volume of hydrosol (three times), according to the method of Ruas et al. [25]. The pentane extracts were concentrated on a Yamato Hitec RE-5 rotary evaporator at room temperature under reduced pressure. Each pentane extract was collected in a vial and concentrated to a minimum volume (100 µL) under nitrogen flow in a benchtop blow-down evaporator system. The hydrolate volatiles were stored at −20 °C until analysis.

### 2.4. Extraction of Volatile Compounds by SPME

The volatile compounds from oregano were also evaluated by headspace solid-phase microextraction (HS-SPME) at room temperature, as detailed by Figueiredo et al. [26]. The volatiles from the plant material were collected using a 100 µm polydimethylsiloxane (PDMS)-coated fiber (Supelco, Bellefonte, PA, USA), manually placed in a special holder (Supelco holder). The SPME fibers were pre-conditioned for 20 min at 250 °C before use, according to the manufacturer’s recommendations. The samples were weighed and placed in glass desiccators (20 cm in diameter) for 1 h to allow homogenization of the internal atmosphere. The fibers were then exposed to the desiccators for 1 h with two replicates per analysis for quantification purposes. The desiccators were washed between analyses, and fiber blanks were performed regularly to identify contaminants in the system and to confirm the absence of compounds that were not representative of the sample under study. After the collection of volatile components, one of the fibers was used for quantitative analysis of the volatile components using gas chromatography with a flame ionization detector (GC-FID), and the other was used for analysis of the chemical composition of each sample using gas chromatography coupled with mass spectrometry (GC-MS).

### 2.5. Identification and Quantification of Volatile Compounds

The chemical characterization of the essential oils and hydrolate volatiles was performed using GC-MS for the identification of the components and GC-FID for quantification of the compounds. A Perkin-Elmer Clarus 400 gas chromatograph (PerkinElmer, Waltham, MA, USA) equipped with two flame ionization detectors and a data acquisition system was used to quantify the compounds under the following experimental conditions: two columns of different polarities were connected to the chromatograph injector, a DB-1 fused silica column (100% dimethylpolysiloxane, 30 m × 0.25 mm i.d., film thickness 0.25 µm; J & W Scientific Inc., Folsom, CA, USA) and a DB-17HT fused silica column (50% (phenyl)-methylpolysiloxane, 30 m × 0.25 mm i.d., film thickness 0.15 µm; J&W Scientific); the carrier gas was hydrogen, set at a linear velocity of 30 cm/s; the partition ratio of the injected volume was 1:40; the oven temperature was programmed to increase from 45 °C (initial temperature) to 175 °C at a rate of 3 °C/min, then to 300 °C at 15 °C/min, where it was maintained in isothermal regime for 10 min, totaling 61.67 min for each analysis; the injector and detector temperatures were 280 and 290 °C, respectively. The quantification of each constituent was calculated by normalizing the peak areas obtained, without the use of correction factors, calculated using the average values of two injections of each sample in accordance with ISO 7609 [27].

Identification of the compounds was achieved on a Perkin-Elmer Clarus 600 gas chromatograph equipped with a DB-1 fused silica column (30 m × 0.25 mm inner diameter, 0.25 µm film thickness; J & W Scientific, Inc., Rancho Cordova, CA, USA) and coupled to a Perkin-Elmer 600T mass spectrometer (software version 5.4.2.1617, Perkin Elmer, Shelton, CT, USA). The experimental parameters were as follows: the injector and detector temperatures were the same as those used for GC/FID; the transfer line and ion source temperatures were 280 °C and 220 °C, respectively; the carrier gas was helium (He), adjusted for a linear velocity of 30 cm/s; the partition ratio was also 1:40; the ionization energy was 70 eV; the scan range was 40–300 u (atomic mass units); and the scan time was 1 s.

The identity of the compounds was assigned by comparing their retention indices with the homologous series of *n*-alkanes (*n*C_8_–*n*C_18_) and the mass spectra from a library developed in the laboratory, as fully detailed in Póvoa et al. [28], based on the analysis of reference essential oils, commercial standards, and laboratory-synthesized compounds.

For the SPME samples, the volatile content was analyzed using the same equipment and the same experimental settings for the essential oils and the hydrolates, but with the following exceptions: the injector was kept at 250 °C and in splitless mode for 1 min for total desorption of the volatiles from the SPME fiber.

### 2.6. Statistical Analysis

The relationship between the percentage composition of essential oils, hydrolate volatiles, and HS-SPME volatile compounds was determined by cluster analysis and principal component analysis (PCA) using the Numerical Taxonomy Multivariate Analysis System (NTSYS PC, version 2.2, Exeter Software, Exeter University, Exeter, UK) [29]. For cluster analysis and PCA, the correlation coefficient was selected as a measure of similarity between samples, and the unweighted pairwise clustering method with arithmetic means (UPGMA) was used to define groups. The degree of correlation was assessed according to the criteria described by Pestana and Gageiro [30], where values between 0.90 and 1.00 are very high; 0.70 and 0.90 are high; 0.40 and 0.70 are moderate; 0.20–0.40, low and <0.20, very low.

## 3. Results and Discussion

### 3.1. Yield and Chemical Composition of the Essential Oils

In the present study, 12 accessions of *Origanum vulgare* subsp. *virens* were evaluated for the yield and chemical composition of EOs obtained by hydrodistillation. Essential oil yield varied markedly among *O. vulgare* accessions, with values ranging from 1.49 to 3.33% (*v*/*w*) (Table 1).

Furthermore, higher yields of essential oil were obtained from the wild-collected samples than from their cultivated counterparts. Sample OV16 contained a more pronounced variation in yield, with the wild accession yielding 3.33%, whereas the cultivated accession yielded 1.61%. These results can be attributed to the distinct edaphoclimatic conditions and the possible level of biotic and abiotic stress faced by the plants. This fact suggests that the greater production of secondary metabolites in wild accessions constitutes an adaptive response for defense and survival [31,32].

The yields found in the present study corroborate those found by Machado et al. [3], who reported a wide range of variations, with values ranging from <0.05 to 3.3% (*v*/*w*) for the same subspecies from different regions of Portugal. Similarly, De Mastro and collaborators [12] studied 25 populations of *O. vulgare* in southern Italy and observed significant variations, with yields varying between 0.96% and 5.10% (*v*/*w*). Both the yield and the essential oil chemical composition are known to be largely influenced by both genetic, physiological, and environmental factors [14]. In addition to variables such as the age of the plant material or the developmental stage, it should be considered that wild plants, generally, even when included in a balanced ecosystem, suffer greater water stress from late spring to the beginning of autumn, which coincides with flowering. Moderate stress (whether biotic or abiotic) tends to promote the production of secondary metabolites, namely the volatiles that constitute part of the secretion accumulated in glandular trichomes, and found in essential oils, as reported by Laftouhi et al. [33] for *Thymus vulgaris, Mentha pulegium*, and *Rosmarinus officinalis.* A comparative study of wild versus cultivated fennel also recorded higher yields from wild plants [34]. However, these differences may vary according to several factors, including genetic, geographic, and environmental factors, but also with the phenological stage and cultivation practices [32].

The chemical characterization of the essential oils allowed the identification of 70 compounds, which correspond to 99–100% of the total composition. The main identified compounds (≥5%) are listed in Table 2, following their elution order on the DB-1 column. The percentage composition of all the compounds identified in each essential oil accession is detailed in Table S1 of the Supplementary Material. The principal classes were oxygen-containing monoterpenes (33–80%) and monoterpene hydrocarbons (8–60%), see Table S1.

A notable chemical variability was observed among the accessions. However, regardless of the material studied, the principal compounds were linalool (0.2–71%), thymol (0.3–39%), carvacrol (0.2–39%), and γ-terpinene (2–31%). This variation can be attributed to factors such as geographic origin. Although these are oregano accessions from a single country (Portugal), the regions in which they were collected are distinct, and the fact that they were from the wild versus cultivated can directly affect these results.

The study of the composition of the essential oil from *Origanum vulgare* is well documented in the literature, revealing and confirming a large chemical variability. Profiles dominated by the phenol-like terpenes, carvacrol and/or thymol, are frequently reported, as was observed by Guo et al. [35] in oregano from China and in several European populations analyzed by Raal et al. [36]. In contrast, the linalool chemotype, although less common, was also found in studies by Lukas et al. [4] in oregano populations from Portugal and Spain, and by Machado et al. [3] in accessions from Portugal, where it is presented as a major constituent with levels exceeding 80%. The diversity also extends to other profiles, such as those rich in caryophyllene oxide and sabinene found in Europe [36] or the γ-terpinene and terpinen-4-ol chemotype identified in samples from Chile by Brito et al. [37]. This broad chemical divergence highlights the importance of characterizing and conserving local genetic resources.

Due to the rich chemical composition of the essential oil from *O. vulgare*, studies demonstrate various biological properties of interest, such as antioxidant, antifungal, antibacterial, insecticidal, anti-inflammatory, healing, antiseptic, and anticancer [8,10,38,39,40]. Furthermore, technologies such as nanoencapsulation are increasingly being developed to optimize these applications. These systems seek to increase the stability of essential oils in aqueous formulations, improve their bioavailability, enable controlled release, minimize toxic effects, and mask their intense aroma to expand their potential uses in the biomedical and biotechnological fields [41].

### 3.2. Chemical Composition of the Hydrolate Volatiles

The volatile compounds obtained from hydrolates of *Origanum vulgare* samples were characterized, and a total of 74 compounds were identified. Although the main constituents were the same as those identified in the essential oils, the percentage distribution in the hydrolate was different (Table 3). The percentage of all the compounds identified for each hydrolate accession are presented in Table S2 of the Supplementary Material.

The oxygen-containing monoterpenes dominated the hydrolate composition (72–96%), followed by monoterpene hydrocarbons (2–22%). This chemical profile is consistent with the greater water solubility and lower volatility of oxygen-containing monoterpenes compared to their hydrocarbon analogs. The presence of monoterpene hydrocarbons can be attributed to the presence of essential oil droplets in the hydrolate aqueous phase.

The hydrolate is often overlooked in phytochemical studies, and the hydrolate volatiles from *Origanum vulgare* have not been extensively explored [16]. To our knowledge, this is the first study to report and compare the chemical composition of hydrolates from different accessions of *O. vulgare* subsp. *virens* from Portugal, highlighting their diversity. Whereas some studies, such as those of Khan et al. [17] and Smiljanic et al. [18] reported the dominance of carvacrol and thymol in the hydrolates from *O. vulgare* L., the present study revealed a greater diversity of compounds.

In addition to finding hydrolates dominated by carvacrol (OV3_C_Hd and OV3_S_Hd), distinct profiles dominated by thymol were also identified in accessions OV21 and OV16 and by linalool in accessions OV20 and OV23. This discovery is of great importance because it demonstrates that the chemical profile of the hydrolate directly reflects the chemotype of the plant from which it originated. These findings allow for greater appreciation of this hydrodistillation co-product in different industrial sectors because hydrolates rich in thymol/carvacrol can be explored for their antimicrobial and antioxidant properties [42], whereas hydrolates rich in linalool would be of great interest to the cosmetic and pharmaceutical industries because of their aromas and anti-inflammatory, analgesic, anxiolytic, and antibacterial properties [43].

### 3.3. Composition of HS-SPME Isolated Volatiles

The evaluation of volatile constituents obtained by HS-SPME, which analyzes the compounds released by the plant before any thermal process, resulted in the identification of 48 organic compounds (Table S3 of the Supplementary Material). As shown in Table 4, the main components (≥5%) of the *O. vulgare* accessions studied were carvacrol (0.8–67%), thymol (6–55%), linalool (1–46%), and γ-terpinene (0.9–19%). A notable difference compared to essential oils and hydrolates was the presence of the sesquiterpene β-caryophyllene as a major component, with a variation of 2–6%.

The predominant chemical classes were oxygen-containing monoterpenes (51–79%), followed by monoterpene (7–34%) and sesquiterpene hydrocarbons (8–21%). In corroboration with other methods, volatile analysis by HS-SPME also allowed the distinction of a linalool chemotype (OV20, OV23) and a chemotype with phenol-like terpene characteristics (OV2, OV3, OV16, OV21), thereby confirming the robustness of these chemical profiles.

The use of the HS-SPME technique is important for comprehensive chemical characterization, as it provides a volatile profile closer to that naturally emitted by the plant, in contrast to hydrodistillation products, which can undergo reactions or rearrangements due to heating. As a rapid, sensitive, and solvent-free method, HS-SPME is ideal for quality control and chemical composition studies [44]. A small quantitative variation can occur because of the variation in volatility and their affinity for the fiber used to absorb the components.

Moreover, the HS-SPME approach also enables the identification of compounds associated with significant biological activities. Although the present work focused on the volatile compounds released *in natura*, studies by Pinto et al. [45] and Hernández- Hernández et al. [46] demonstrated that the constituents identified in the vapors of *O. vulgare* essential oil exerted a remarkable antibacterial effect. These findings highlight that the chemical characterization obtained by HS-SPME is not restricted to quality control or compositional studies but also provides valuable information on the functional potential of volatile compounds.

A finding that demonstrates an important difference between diverse volatile isolation procedures is the higher percentage of β-caryophyllene observed with HS-SPME. These results corroborate those found by Karami-Osboo et al. [44], who also reported a higher percentage of β-caryophyllene by HS-SPME (18%) than in the essential oil (6%) when they compared the essential oil and volatile oil analyses by HS-SPME for *Thymus daenensis*. This fact suggests that β-caryophyllene might be more sensitive to heat, being partially degraded or hardly carried over during distillation. Furthermore, the scarcity of comparative studies using HS-SPME in *O. vulgare* subsp. *virens* reinforces the novelty and importance of the data presented here.

### 3.4. Cluster Analysis

**Figure 1:**
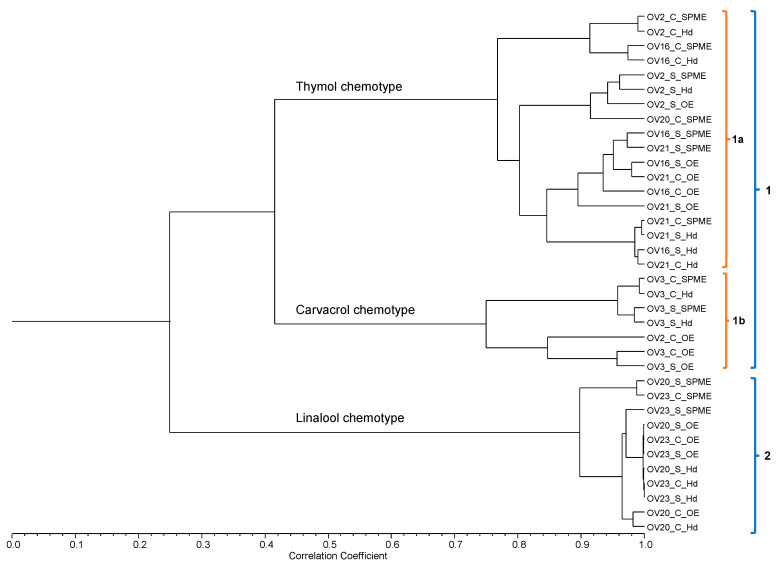
Dendrogram obtained by cluster analysis of the percentage composition of the essential oils, the hydrolate and HS-SPME volatiles, isolated from 12 *Origanum vulgare* subsp. *virens* samples, based on correlation and using the unweighted pair-group method with arithmetic average (UPGMA). 1 and 2: Main clusters. 1a and 1b: Subclusters. For samples codes, see Table 1.

The relationship between chemical compositions among all the samples studied by the different techniques was assessed through hierarchical clustering using the correlation coefficient as a measure of similarity. The resulting dendrogram (Figure 1) revealed the formation of two main clusters (Group 1 and Group 2) and two subclusters (Group 1a and Group 1b).

A low correlation coefficient (≈0.25) was obtained for Clusters 1 and 2, indicating major chemical divergence between them. This fact can be attributed to the proportions of some major compounds because the identified compounds were generally similar.

Cluster 2 consisted of the linalool chemotype, dominant in accessions OV20 (Serpa) and OV23 (Moura) in all the analyses. The high similarity between them (correlation coefficient of ≈0.90) highlights a stable chemical profile dominated by linalool, whose levels ranged from 35% to 77% in all the samples in this group (Table 5). These results corroborate those found by Machado et al. [3], who evaluated the essential oil from *O. vulgare* from various regions of Portugal, including Serpa and Moura. In that study, oregano from these two origins was grouped into subcluster Ia1, which was defined by the dominance of linalool, the same chemotype found in the present study.

Cluster 1, which represents the chemotype of samples dominated by phenol-like terpenes, was more heterogeneous, being subdivided into two main subgroups. Cluster 1a was characterized by the dominance of thymol, attaining up to 82% (Table 5), grouping accessions OV16 (Sousel), OV21 (Alandroal), and OV2 (Estremoz). Subgroup 1b was defined by the predominance of carvacrol (with levels up to 93%) and its precursor γ-terpinene, which are represented by accession OV3 (Elvas) and sample OV2_C_OE (Table 5).

This general profile is consistent with the findings of Machado et al. [3]. However, a notable divergence was observed for accession OV2 (Estremoz). In the present study, a profile containing predominantly thymol was observed for this accession, whereas the Estremoz accessions (Es1 and Es2) were classified as a carvacrol chemotype in the studies by Machado et al. [3]. According to Figueiredo et al. [14], this variation within the same geographic origin can be attributed to several factors, such as genetics, climatic conditions, the influence of growing conditions and the year and the harvest phase, which can influence the biosynthetic pathway and favor one isomer over another. Furthermore, this variability in the chemical composition of *O. vulgare* is extensively discussed in the literature. Studies by Raal et al. [36] demonstrated that the essential oil profile of *O. vulgare* varies significantly with subspecies, geographic origin, and growth stage. In another study, Lukas et al. [4] characterized multiple chemotypes across European populations and showed that both genetic background and environmental factors (e.g., latitude, altitude, and local climate) influence the monoterpenes/sesquiterpenes ratio and the relative abundance of major constituents.

Castilho et al. [47] reported significant differences in the relative amounts of thymol, γ-terpinene, and *p*-cymene in oregano populations from Madeira Island, attributing these patterns mainly to local environmental conditions. Complementarily, Vale-Silva et al. [48] demonstrated that the percentage of major constituents such as carvacrol and γ-terpinene varied considerably among samples, which directly influenced the antifungal activity of *O. vulgare* essential oils.

Although the accessions evaluated using the different methods predominantly fell within the same group, some more discrepant samples were observed. A chemotype difference was observed in accession OV20 (Serpa), where most of the samples belonged to the linalool chemotype. However, HS-SPME analysis revealed a thymol-dominated profile in the cultivated species (OV20_C_SPME). Another notable variation occurred in accession OV2 (Estremoz), where the essential oil of the cultivated species (OV2_C_OE) was dominated by γ-terpinene, in contrast to the other samples of the same accession, which contained thymol as the principal compound. Novak et al. [49] also reported that thymol significantly decreased while carvacrol increased in *Origanum* species OEs with increasing temperatures during flowering. The average temperature of the climatological normal for the months of May and June, during *Origanum* flowering, in Estremoz (location of wild OV2 harvest) is lower than that of Elvas where it was cultivated (OV2_C) [50]; this may partially explain the higher carvacrol levels obtained from all the extracts of the wild samples versus the cultivated samples.

### 3.5. Principal Components Analysis (PCA)

**Figure 2:**
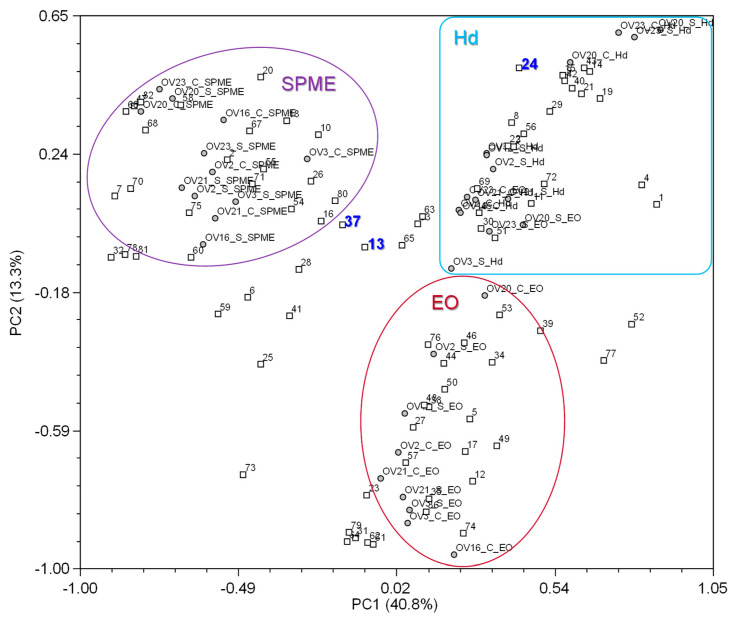
Graph obtained by PCA of the percentage composition of the essential oils (EO in dark red), the hydrolate volatiles (Hd in light blue) and HS-SPME volatiles (SPME in purple), isolated from 12 *Origanum vulgare* subsp. *virens* samples, based on correlation. Carvacrol (number label 13), thymol (number label 37), and linalool (number label 24) are highlighted in bold. For other compounds’ number label, see Table 2, Table 3, Table 4 and Table 5 and Tables S1–S3. For samples codes, see Table 1.

Principal component analysis (PCA) was performed to identify the distribution of variance among the variables. Thirty-five components were needed to explain the distribution of accessions and volatile compounds, with the three main components only explaining 54% of the variability, see Figure 2.

In the overall PCA, the two-dimensional coordinate system of the first and second principal components shows that the distribution of accessions was mainly influenced by the extraction method, as depicted in Figure 2, with (1) the essential oils isolated by hydrodistillation highlighted in the dark red circle, the (2) the volatiles obtained from the hydrolate, by liquid/liquid extraction, in the light blue circle, and (3) the headspace volatiles obtained by HS-SPME in the purple circle.

In the general analysis, the compounds’ characteristic of the chemotypes, thymol (number label 37) and carvacrol (number label 13), were not very important (closer to the center of the projection), although linalool (number label 24) was an important compound for projection. Other compounds unique to the extracts were fundamental for the definition of the PCA (labels furthest from the center of the projection). Among these are, for example, 1-octen-3-ol and 3-octanone (labels # 1 and 4 in Figure 2 and Tables S1 and S2), which were not detected in HS-SPME, and contributed significantly to the accessions grouping in PCA. Likewise, thuja-2,4(10)-diene (label # 36 in Figure 2 and Table S1), which was only detected in the EOs, contributed significantly to the accession grouping in PCA. Within each extraction procedure, the samples were grouped by their chemotype, as shown in cluster analysis, Figure 1, and in PCA graphs in Figures S1–S3.

Several works with oregano essential oil used multivariate analysis, like PCA, to identify the differences among the essential oil components. Novák et al. [51] analyzed the essential oil components obtained from various *Origanum* species from Budapest. PCA also enabled the grouping of EOs obtained from Montenegro *Origanum vulgare* in distinct chemotypes as described by Stešević et al. [52]. The chemical variations among different populations of red oregano from Albania (*Origanum vulgare* L. subsp. *vulgare*) were also studied by Kadiasi et al. [53], with PCA contributing to identify the variance distribution among the variables, specifically essential oil compounds obtained from populations collected at different locations.

## 4. Conclusions

This study demonstrated the remarkable chemical diversity of accessions of *Origanum vulgare* subsp. *virens* in South Portugal and revealed that the perceived chemical profile can be influenced by the analytical method. Therefore, chemical characterization should not be limited to the essential oil because the volatile components of the hydrolate and those obtained by HS-SPME offer complementary and valuable chemical information.

The identification of chemotypes reinforces the importance of conserving this national germplasm, both in gene banks (ex situ) and in their natural habitats (in situ), to preserve this variability. From an industrial perspective, this multifaceted knowledge can contribute to the detection of both plant and volatiles adulteration and allows for the valorization of resources. Rather than merely selecting a plant, the most suitable matrix for the desired purpose can be achieved according to the intended application, such as an essential oil, to obtain high concentrations of phenol-like terpenes with antimicrobial potential, or a hydrolate of a linalool chemotype, rich in this alcohol, for applications in the cosmetics industry.

Therefore, detailed knowledge of the chemical profiles of the different matrices will allow producers to make wiser and more targeted use of each accession and align production with specific market demands.

## Figures and Tables

**Table 1:** Samples of *O. vulgare* studied, with indication of the original collection site (wild, S), BPGV accession code, and the EO yield. The cultivated plants (C) were obtained from the ESBE/IPP Experimental Field.

Access	BPGV	Origin: Alentejo (Municipality, District, Country)	EO Yield (%, *v*/*w*)	Code
OV2_wild	BPGV19646	Estremoz, Évora, Portugal	1.97	OV2_S
OV2_cultivated	BPGV19646	Estremoz, Évora, Portugal	1.79	OV2_C
OV3_wild	BPGV19647	Elvas, Portalegre, Portugal	2.70	OV3_S
OV3_cultivated	BPGV19647	Elvas, Portalegre, Portugal	1.87	OV3_C
OV16_wild	BPGV27705	Sousel, Portalegre, Portugal	3.33	OV16_S
OV16_cultivated	BPGV27705	Sousel, Portalegre, Portugal	1.61	OV16_C
OV20_wild	BPGV27709	Serpa, Beja, Portugal	2.08	OV20_S
OV20_cultivated	BPGV27709	Serpa, Beja, Portugal	1.49	OV20_C
OV21_wild	BPGV28151	Alandroal, Évora, Portugal	1.88	OV21_S
OV21_cultivated	BPGV28151	Alandroal, Évora, Portugal	2.09	OV21_C
OV23_wild	BPGV28153	Moura, Beja, Portugal	1.79	OV23_S
OV23_cultivated	BPGV28153	Moura, Beja, Portugal	1.72	OV23_C

BPGV (Banco Português de Germoplasma Vegetal): Portuguese Plant Germplasm Bank (storage code in BPGV).

**Table 2:** Composition of the main components (≥5%) of essential oils extracted from *Origanum vulgare*.

#	Compounds	RI	OV2 _S_ OE	OV2 _C_ OE	OV3 _S_ OE	OV3 _C_ OE	OV16 _S_ OE	OV16 _C_ OE	OV20 _S_ OE	OV20 _C_ OE	OV21 _S_ OE	OV21 _C_ OE	OV23 _S_ OE	OV23 _C_ OE
31	*p*-Cymene	1003	4.2	8.7	6.9	8.9	5.7	5.7	0.6	3.8	8.8	7.9	1.7	0.8
17	*cis*-β-Ocimene	1017	3.6	3.0	4.7	2.4	3.1	5.0	4.6	6.4	7.9	4.4	4.6	3.0
79	γ-Terpinene	1035	18.7	29.1	31.4	24.3	27.4	22.2	2.1	8.9	30.4	25.0	2.6	2.1
24	Linalool	1074	20.5	2.1	0.3	0.4	6.3	1.8	70.0	48.5	0.3	0.2	69.6	71.1
37	Thymol	1275	24.6	17.7	4.2	0.3	34.1	27.4	3.3	12.4	24.3	39.0	5.6	6.2
13	Carvacrol	1286	7.3	17.5	29.2	38.8	3.5	12.9	1.5	0.2	0.3	0.3	1.4	0.4

#: Number used in PCA. RI: Laboratory-calculated retention index for C_9_ to C_17_ *n*-alkanes on the DB-1 column.

**Table 3:** Chemical composition of the main components (≥5%) of the volatile compounds from *Origanum vulgare* hydrolates.

#	Compounds	RI	OV2 _S_ Hd	OV2 _C_ Hd	OV3 _S_ Hd	OV3 _C_ Hd	OV16 _S_ Hd	OV16 _C_ Hd	OV20 _S_ Hd	OV20 _C_ Hd	OV21 _S_ Hd	OV21 _C_ Hd	OV23 _S_ Hd	OV23 _C_ Hd
79	γ-Terpinene	1035	9.4	7.9	13.3	1.4	6.5	4.9	0.7	3.1	11.9	5.9	1.1	0.9
24	Linalool	1074	21.8	2.3	0.4	0.3	5.5	1.5	76.9	56.1	0.5	0.2	72.6	76.7
37	Thymol	1275	37.9	37.0	8.4	0.8	66.7	49.4	3.2	19.6	63.9	82.3	7.2	6.4
13	Carvacrol	1286	13.0	37.6	60.0	92.5	8.5	30.0	1.4	0.3	0.7	0.6	2.0	0.4

#: Number used in PCA. RI: Laboratory-calculated retention index for C_9_ to C_17_ *n*-alkanes on the DB-1 column.

**Table 4:** Chemical composition of the main (≥5%) volatile components obtained from *Origanum vulgare* by HS-SPME.

#	Compounds	RI	OV2 _S_ SPME	OV2 _C_ SPME	OV3 _S_ SPME	OV3 _C_ SPME	OV16_S_ SPME	OV16 _C_ SPME	OV20 _S_ SPME	OV20 _C_ SPME	OV21 _S_ SPME	OV21 _C_ SPME	OV23 _S_ SPME	OV23 _C_ SPME
79	γ-Terpinene	1035	13.6	9.9	14.6	6.3	19.3	6.5	1.4	4.2	14.2	12.1	2.1	0.9
24	Linalool	1074	13.2	2.3	1.1	1.6	6.8	2.1	39.2	24.0	2.8	1.1	46.4	34.7
37	Thymol	1275	26.4	28	10.7	5.5	34.7	47.9	17.6	30.3	36.8	54.8	9.8	21.8
13	Carvacrol	1286	11.3	32.8	41.8	66.7	5.1	17.5	9.1	4.1	8.7	0.8	8.2	11.2
68	β-Caryophyllene	1414	5.1	5.0	4.0	2.3	4.7	2.5	5.9	5.6	5.0	3.4	5.5	5.6

#: Number used in PCA. RI: Laboratory-calculated retention index for C_9_ to C_17_ *n*-alkanes on the DB-1 column.

**Table 5:** Minimum and maximum percentage range of the main components (≥2% in at least one sample) of essential oils, hydrolate, and HS-SPME volatiles, isolated from *Origanum vulgare* subsp. *virens* accessions grouped according to cluster analysis.

#			Cluster 1				Cluster 2	
	Components	RI	Cluster 1a		Cluster 1b			
			Min	Max	Min	Max	Min	Max
64	α-Thujene	924	0.1	2.4	t	2.2	t	0.6
73	β-Myrcene	975	0.3	3.1	0.1	3.1	0.1	3.1
62	α-Terpinene	1002	0.5	4.1	0.1	3.6	t	1.1
31	*p*-Cimene	1003	1.2	8.8	0.6	8.9	0.2	3.8
17	*cis*-β-Ocimene	1017	0.7	7.9	0.1	4.7	1.1	6.4
79	γ-Terpinene	1035	4.2	30.4	1.4	31.4	0.7	8.9
43	*trans*-Linalool oxide (furanoid)	1059	t	0.5	0.1	0.1	0.3	2.7
24	Linalool	1074	0.2	24.0	0.3	2.1	34.7	76.9
34	Terpinen-4-ol	1148	0.2	2.8	0.2	1.2	0.1	0.7
28	Methyl thymol	1210	t	5.2	t	1.7	0.2	2.0
25	Methyl carvacrol	1224	t	3.3	0.9	7.1	0.3	2.0
37	Thymol	1275	24.3	82.3	0.3	17.7	3.2	21.8
13	Carvacrol	1286	0.3	37.6	17.6	92.5	0.2	11.2
68	β-Caryophyllene	1414	0.8	5.6	0.2	4.0	1.7	5.9
20	Germacrene D	1474	0.2	3.6	t	1.1	1.1	4.2
10	Bicyclogermacrene	1487	0.3	1.6	t	1.1	0.7	2.3
66	β-Bisabolene	1500	0.1	4.2	0.1	2.5	0.3	3.9
	**Identification (%)**		96.0	100.0	97.6	100.0	96.4	100.0
	**Grouped Components**							
	Monoterpene hydrocarbons		9.6	60.4	2.4	55.7	3.0	24.5
	Oxygen-containing monoterpenes		33.2	86.1	38.6	96.3	65.3	88.5
	Sesquiterpene hydrocarbons		2.3	20.6	0.4	12.3	5.4	20.7
	Oxygen-containing sesquiterpenes		0.1	1.4	t	0.8	0.3	1.0
	Phenylpropanoids		t	0.4	t	0.2	t	0.1
	Others		t	1.9	t	0.8	0.3	1.7

#: Number used in PCA. RI: Laboratory-calculated retention index for C_9_–C_16_ *n*-alkanes on the DB-1 column. Min: minimum. Max: maximum. t: traces (<0.05%).

## Data Availability

The original contributions presented in the study are included in the article/supplementary material, further inquiries can be directed to the corresponding author.

## Supplementary Materials

The following supporting information can be downloaded at: https://www.mdpi.com/article/10.3390/foods14244175/s1, Table S1. Percentage composition of the essential oil obtained by hydrodistillation from *Origanum vulgare* subsp. *virens* accessions from Estremoz, Elvas, Sousel, Serpa, Alandroal and Moura. For samples codes, see Table 1. Table S2. Percentage composition of the hydrolate volatiles of *Origanum vulgare* subsp. *virens* accessions from Estremoz, Elvas, Sousel, Serpa, Alandroal, and Moura. For samples codes, see Table 1. Table S3. Percentage composition of volatile compounds obtained by HS-SPME from *Origanum vulgare* subsp. *virens* accessions from Estremoz, Elvas, Sousel, Serpa, Alandroal and Moura. For samples codes, see Table 1. Figure S1. Graph obtained by PCA of the percentage composition of the essential oils (EO), isolated from 12 *Origanum vulgare* subsp. *virens* samples, based on correlation. Main compounds/chemotypes: carvacrol (number label 13, light blue), thymol (number label 37, purple) and linalool (number label 24, dark red). For other compounds number label, see Table 2, Table 3, Table 4 and Table 5 and Tables S1–S3. For samples codes, see Table 1. Figure S2. Graph obtained by PCA of the percentage composition of the hydrolate volatiles (Hd), isolated from 12 *Origanum vulgare* subsp. *virens* samples, based on correlation. Main compounds/chemotypes: carvacrol (13, light blue), thymol (37, purple) and linalool (24, dark red). For other compounds number label, see Table 2, Table 3, Table 4 and Table 5 and Tables S1–S3. For samples codes, see Table 1. Figure S3. Graph obtained by PCA of the percentage composition of HS-SPME volatiles (SPME), isolated from 12 *Origanum vulgare* subsp. *virens* samples, based on correlation. Main compounds/chemotypes: carvacrol (13, light blue), thymol (37, purple) and linalool (24, dark red). For other compounds number label, see Table 2, Table 3, Table 4 and Table 5 and Tables S1–S3. For samples codes, see Table 1.
