# Supplementary material for: Comparative Evaluation of the Volatile Profile of the Essential Oil, the Hydrolate and the Plant Material from Origanum vulgare subsp. virens Grown in Portugal
Source: Foods. 2025 Dec 5;14(24):4175. doi: 10.3390/foods14244175 (PMC12731851; doi:10.3390/foods14244175)
Supplement: Supplementary file 1 [file foods-14-04175-s001.zip › foods-3995334-supplementary.pdf]

Article

Comparative Evaluation of the Volatile Profile of the Essential Oil, the Hydrolate and the Plant Material from *Origanum vulgare* subsp. *virens* Grown in Portugal

Carolina Salles Freire[1], Maria das Graças Cardoso[1], Orlanda Póvoa[2], Noémia Farinha[2], David Lee Nelson[3], Alexandra M. Machado[4], Ana Cristina Figueiredo[4]*

1 Chemistry Department, Federal University of Lavras (UFLA), Lavras, Minas Gerais, Brazil; carolina.freire2@estudante.ufla.br, mcardoso@ufla.br

2 VALORIZA - Centro de Investigação para a Valorização de Recursos Endógenos, Instituto Politécnico de Portalegre, Praça do Município 11, 7300-110 Portalegre, Portugal; opovoa@ipportalegre.pt, nfarinha@ipportalegre.pt

3 Postgraduate Program in Biofuels, Federal University of the Jequitinhonha and Mucuri Valleys, Diamantina, Minas Gerais, Brazil; dleenelson@gmail.com

4 Centre for Ecology, Evolution and Environmental Changes (CE3C) & Global Change and Sustainability Institute (CHANGE), Faculdade de Ciências da Universidade de Lisboa, Biotecnologia Vegetal, DBio, Campo Grande, 1749-016 Lisboa, Portugal; ampmachado@fc.ul.pt, acsf@fc.ul.pt

* Correspondence: ACF acsf@fc.ul.pt; CSF carolina.freire2@estudante.ufla.br

Table S1. Percentage composition of the essential oil obtained by hydrodistillation from *Origanum vulgare* subsp. *virens* accessions from Estremoz, Elvas, Sousel, Serpa, Alandroal and Moura. For samples codes, see Table 1.

#	Components	RI	OV2_S	OV2_C	OV3_S	OV3_C	OV16_S	OV16_C	OV20_S	OV20_C	OV21_S	OV21_C	OV23_S	OV23_C
49	Tricyclene	921		t	t	t	t	t	t	t	t	t	t	t
64	α-Thujene	924	1.0	1.6	2.2	1.8	1.8	2.4	0.3	0.6	2.0	2.0	0.3	0.2
61	α-Pinene	930	0.4	0.8	0.9	0.9	0.7	0.8	0.1	0.3	0.8	0.8	0.2	0.1
36	Thuja-2,4(10)-diene	940	t	t	t	t		t		t	t	t		
12	Camphene	938	0.1	0.1	0.2	0.2	0.1	0.1	0.1	0.2	0.2	0.1	0.2	0.1
32	Sabinene	958	0.2	0.2	0.2	0.2	0.2	0.2			0.3	0.2		
1	1-Octen-3-ol	961	0.2	0.2	0.2	0.3	0.2	0.2	0.5	0.6	0.3	0.2	0.4	0.4
4	3-Octanone	961	0.2	0.1	0.1	0.2		0.2	0.5	0.6	0.3	0.2	0.4	0.4
75	β-Pinene	963	0.2	0.2	0.2	0.2	0.2	0.2			0.3	0.2	0.4	0.4
73	β-Myrcene	975	1.7	2.8	3.1	3.0	2.6	2.3	0.7	1.4	3.1	2.9	0.9	0.6
60	α-Phellandrene	995	0.2	0.4	0.4	0.3	0.3	0.3	t	0.1	0.4	0.4	0.1	0.1
80	δ-3-Carene	1000	t	0.1	0.1	0.1	0.1	0.1	t	t	0.1	0.1	t	t
62	α-Terpinene	1002	2.1	3.6	3.6	3.2	3.7	3.2	0.4	1.1	4.1	3.8	0.5	0.3
31	*p*-Cymene	1003	4.2	8.7	6.9	8.9	5.7	5.7	0.6	3.8	8.8	7.9	1.7	0.8
74	β-Phellandrene	1005	0.1	0.3	0.3	0.3	0.3	0.3	t	0.1	0.3	0.3	0.1	t
23	Limonene	1009	0.2	0.4	0.4	0.3	0.4	0.3	0.1	0.2	0.4	0.4	0.1	0.1
17	*cis*-β-Ocimene	1017	3.6	3.0	4.7	2.4	3.1	5.0	4.6	6.4	7.9	4.4	4.6	3.0
48	*trans*-β-Ocimene	1027	0.6	0.5	0.7	0.4	0.5	0.8	0.7	1.0	0.9	0.6	0.8	0.5
79	γ-Terpinene	1035	18.7	29.1	31.4	24.3	27.4	22.2	2.1	8.9	30.4	25.0	2.6	2.1
45	*trans*-Sabinene hydrate	1037	t	t	t	t	t	t	0.1	t	t	t	t	t
14	*cis*-Linalool oxide (furanoid)	1045	0.1						0.1	0.1	t		0.2	0.1
2	2,5-Dimethyl styrene	1059		t		0.1	t	t			t	t		
43	*trans*-Linalool oxide (furanoid)	1059							0.3	0.3			0.3	0.4
35	Terpinolene	1064	0.1	0.1	0.2	0.2	0.1	0.4	0.1	0.1	0.1	0.1	0.1	t
16	*cis*-Sabinene hydrate	1066	0.1	t	t	t	0.1	0.8	0.2	0.1	0.1	t	t	t
24	Linalool	1074	20.5	2.1	0.3	0.4	6.3	1.8	70.0	48.5	0.3	0.2	69.6	71.1
44	*trans*-*p*-2-Menthen-1-ol	1099	t	t	t	t	t	0.2			t	t	t	t
6	*allo*-Ocimene	1100	0.3	0.2	0.2	0.1	0.1	0.3	0.2	0.3	0.3	0.2	0.2	0.1
11	Borneol	1134	0.3	0.2	0.3	0.2	0.2	0.2	0.3	0.2	0.3	0.2	0.3	0.1
34	Terpinen-4-ol	1148	0.6	0.8	0.8	1.0	0.7	2.8	0.4	0.4	0.5	0.6	0.2	0.2
27	Methyl salicylate	1159	t	t	0.1	0.1		t	t	t	t	t	t	t
63	α-Terpineol	1159	0.1	0.1	0.1	0.1	0.8	0.2	0.1	0.1	0.1	0.1	0.2	0.1
29	Nerol	1206							t	t			0.1	t
28	Methyl thymol	1210	1.4	1.4	0.5	0.1	0.5	0.1	0.3	2.0	5.2	1.8	0.5	0.7
25	Methyl carvacrol	1224	1.9	1.6	3.2	7.1	0.9	0.2	0.5	0.9	2.1	2.5	0.9	0.4
19	Geraniol	1236	0.1		t	t			0.1	0.1			0.1	0.1
30	*p*-Cymen-7-ol	1265	t	t	t	t	t	t			t	t		
37	Thymol	1275	24.6	17.7	4.2	0.3	34.1	27.4	3.3	12.4	24.3	39.0	5.6	6.2
13	Carvacrol	1286	7.3	17.5	29.2	38.8	3.5	12.9	1.5	0.2	0.3	0.3	1.4	0.4
18	Eugenol	1327	t	t		t	t		t		t			
38	Thymol acetate	1327				0.1		0.1		t	t	0.1		t
82	δ-Elemene	1332	0.3	t	0.1	t	t	t	0.2	0.1	0.1	t	0.1	0.1
55	α-Cubebene	1345	t	0.1	t		t	0.1	0.1	t	t	t	t	t
65	α-Ylangene	1371	t	t	t	t	t	t	t	t	t	t	0.1	0.1
54	α-Copaene	1375	0.1	t	t	t	t	0.1	t	t	t	t	t	t
67	β-Bourbonene	1379	0.1	t	0.1	0.1	t	0.1	0.2	0.2	0.1	t	0.1	0.2

#	Components	RI	OV2 _S	OV2 _C	OV3 _S	OV3 _C	OV16 _S	OV16 _C	OV20 _S	OV20 _C	OV21 _S	OV21 _C	OV23 _S	OV23 _C
72	β-Elemene	1388	0.1				t		0.1	0.1			0.1	0.1
68	β-Caryophyllene	1414	2.4	2.4	1.5	1.5	1.6	1.7	3.0	2.7	1.7	1.8	2.4	2.6
70	β-Copaene	1426	0.1	0.1	0.1	0.1	t	0.1	0.1	0.1	t	0.1	0.1	0.1
7	Aromadendrene	1428	0.3	0.3	0.3	0.2	0.2	0.2	0.1	0.1	0.3	0.4	0.1	0.1
57	α-Guaiene	1427		t			t	t			t	t		
58	α-Humulene	1447	0.4	0.3	0.2	0.1	0.2	0.3	0.5	0.5	0.2	0.3	0.4	0.5
5	*allo*-Aromadendrene	1456	0.1	t	0.1	t	t	0.1	0.1	t	t	0.1	t	t
78	γ-Muurolene	1469	0.2	0.2	0.2	0.2	0.2	0.2	t	0.1	0.2	0.2	t	0.1
20	Germacrene D	1474	1.0	0.5	0.4	0.2	0.6	0.9	2.1	1.8	0.4	0.5	1.3	3.0
76	β-Selinene	1476	0.1	t	t	t	t	t			t	t		t
50	Valencene	1484	t	t	t	0.1	t	t	t		t	t	t	t
10	Bicyclogermacrene	1487	1.3	0.6	0.6	0.3	0.6	0.5	2.3	1.1	0.8	0.8	0.8	1.4
59	α-Muurolene	1494	0.1	0.1	t	0.1	0.1	0.1	t	t	t	0.1	t	t
39	*trans,trans*-α-Farnesene	1500	0.4	0.3	0.2	0.3	0.2	0.8	0.4	0.3	0.1	0.2	0.3	0.5
66	β-Bisabolene	1500	0.4	0.3	0.2	0.3	0.2	0.8	0.4	0.3	0.2	0.2	0.3	0.5
77	γ-Cadinene	1500	0.4	0.3	0.2	0.3	0.2	0.8	0.4	0.3	0.2	0.2	0.3	0.5
41	*trans*-Calamenene	1505			t	t		t	t		t		t	t
81	δ-Cadinene	1505	0.3	0.3	0.3	0.3	0.6	0.4	0.2	0.2	0.2	0.3	0.1	0.2
52	α-Cadinene	1529	t	t	t	t	t	t	t	t	t			t
46	*trans*-α-Bisabolene	1536	0.1	t	0.1	t	t	0.1	t	t	t		t	t
33	Spathulenol	1551	0.4	0.2	0.2	0.1	0.2	0.2	0.4	0.3	0.3	0.1	0.2	0.2
69	β-Caryophyllene oxide	1561	0.1	0.1	0.1	t	0.1	0.1	0.2	0.2	0.1	t	0.2	0.1
51	Viridiflorol	1569	t	t			t	t	0.1	t	t			t
53	α-Cadinol	1630	0.1	0.1			0.1	0.1	0.3	t	0.1		0.1	0.1
	**% Identification**		99.9	100.0	99.6	99.8	99.7	99.4	99.4	99.4	99.2	99.9	100.0	99.4
	**Grouped Components**													
	Monoterpene hydrocarbons		33.7	52.1	55.7	46.8	47.3	44.6	10.0	24.5	60.4	49.4	12.8	8.4
	Oxygen-containing monoterpenes		57.0	41.4	38.6	48.1	47.1	46.7	77.2	65.3	33.2	44.8	79.4	79.8
	Sesquiterpene hydrocarbons		8.2	5.8	4.6	4.1	4.7	7.3	10.2	7.9	4.5	5.2	6.5	10.0
	Oxygen-containing sesquiterpenes		0.6	0.4	0.3	0.1	0.4	0.4	1.0	0.5	0.5	0.1	0.5	0.4
	Others		0.4	0.3	0.4	0.7	0.2	0.4	1.0	1.2	0.6	0.4	0.8	0.8

#: Number used in PCA analysis. RI: Laboratory-calculated retention index for C_9 to C_{17} *n*-alkanes on the DB-1 column. t: traces (<0.05%)

Table S2. Percentage composition of the hydrolate volatiles of *Origanum vulgare* subsp. *virens* accessions from Estremoz, Elvas, Sousel, Serpa, Alandroal, and Moura. For samples codes, see Table 1.

#	Components	RI	OV2 _S	OV2 _C	OV3 _S	OV3 _C	OV16 _S	OV16 _C	OV20 _S	OV20 _C	OV21 _S	OV21 _C	OV23 _S	OV23 _C
40	*trans*-2-Hexenal	866	0.1	t	t	0.1	0.1	t	0.1	0.2	0.2	0.1	0.1	0.1
49	Tricyclene	921		t					t					
64	α-Thujene	924	0.2	0.1	0.4	t	0.2	0.3	0.1	0.2	0.4	0.2	0.1	t
61	α-Pinene	930	0.1	t	0.2	t	0.2	0.1	t	0.1	0.2	0.1	t	t
12	Camphene	938	t	t	t	t	t	t	t	t	t	t	t	t
1	1-Octen-3-ol	961	0.2	0.2	0.2	0.6	0.2	0.1	0.6	0.4	0.6	0.2	0.6	0.7
4	3-Octanone	961	0.2	0.2	0.2		0.2	0.1	0.6	0.4	0.6	0.2	0.6	0.7
75	β-Pinene	963	0.2	0.2	0.2		0.2	0.1	t	0.4	0.6	0.2		
3	3-Octanol	974	t	0.3					0.1	0.2	0.5	t	0.2	0.1
73	β-Myrcene	975	0.7	0.3	1.0	0.1	0.4	0.4	0.1	0.2	0.5	0.5	0.2	0.2
60	α-Phellandrene	995	0.1	0.1	0.1	t	0.1	t	t	0.1	0.1	0.1	0.1	t
80	δ-3-Carene	1000	0.2		t	0.1	0.1	0.3	0.1	0.2	0.1	0.1	0.1	t
8	Benzene acetaldehyde	1002				0.1							0.1	0.1
62	α-Terpinene	1002	0.9	0.8	1.3	0.1	0.7	0.6	0.1	0.3	1.3	0.7	0.1	0.1
31	*p*-Cymene	1003	1.6	1.9	2.3	0.6	1.2	1.2	0.2	1.2	3.5	1.8	0.7	0.3
74	β-Phellandrene	1005	0.1	0.1	0.2	t	0.1	0.1	t	t	0.1	0.1	t	t
23	Limonene	1009	0.1	0.1	0.1	t	0.1	0.1	t	0.1	0.2	0.1	0.1	t
17	*cis*-β-Ocimene	1017	1.8	0.8	1.9	0.1	0.7	1.1	1.6	2.2	3.0	1.0	2.0	1.3
48	*trans*-β-Ocimene	1027	0.3	0.1	0.3	t	0.1	0.2	0.3	0.4	0.4	0.2	0.4	0.2
79	γ-Terpinene	1035	9.4	7.9	13.3	1.4	6.5	4.9	0.7	3.1	11.9	5.9	1.1	0.9
45	*trans*-Sabinene hydrate	1037	t	t	t	t	t	0.3	0.1	t	t	t	t	t
14	*cis*-Linalool oxide (furanoid)	1045	0.2	t	t	0.1	0.1	t	0.9	0.6	0.1	t	1.1	0.7
2	2,5-Dimethyl styrene	1059		t	t						t			
43	*trans*-Linalool oxide (furanoid)	1059	0.5			0.1	0.2	0.1	2.7	2.0		t	2.4	2.5
35	Terpinolene	1064	t	t	0.1	t	t	0.1	t	t	t	t	t	t
16	*cis*-Sabinene hydrate	1066	0.1	t	t	t	0.2	0.7	0.2	0.1	0.1	0.1	t	t
24	Linalool	1074	21.8	2.3	0.4	0.3	5.5	1.5	76.9	56.1	0.5	0.2	72.6	76.7
44	*trans*-*p*-2-menthen-1-ol	1099	t		t	t	t	0.1	t	t	t	t		
6	*allo*-Ocimene	1100	0.1	t	0.2	t	t	0.1	0.1	0.1	0.1	0.1	0.1	t
42	*trans*-Limonene oxide	1119					t		t	t		t	t	t
11	Borneol	1134	0.3	0.3	0.3	0.2	0.3	0.2	0.4	0.4	0.6	0.2	0.4	0.2
15	*cis*-Linalool oxide (pyranoid)	1132							0.1	0.1			0.1	0.1
34	Terpinen-4-ol	1148	0.8	1.2	1.1	1.2	0.9	2.8	0.6	0.7	0.7	0.7	0.3	0.3
63	α-Terpineol	1159	0.2	0.1	0.1	0.1	0.8	0.3	0.3	0.3	0.1	0.1	0.3	0.3
29	Nerol	1206							0.1	0.1			0.1	t
28	Methyl thymol	1210	1.1	1.1	0.4	t	0.3	t	0.2	1.5	3.5	0.7	0.4	0.4
25	Methyl carvacrol	1224	1.4	1.3	2.8	0.9	0.5	t	0.3	0.7	1.5	1.0	0.7	0.3
19	Geraniol	1236	0.1						0.2	0.2			0.2	0.2
22	Indole	1251	t	t	t		0.1	0.1	0.1	t	t	t	t	t
30	*p*-Cymen-7-ol	1265	t	t	t	0.1	t	t	t	t	t	0.1	t	t
37	Thymol	1275	37.9	37.0	8.4	0.8	66.7	49.4	3.2	19.6	63.9	82.3	7.2	6.4
13	Carvacrol	1286	13.0	37.6	60.0	92.5	8.5	30.0	1.4	0.3	0.7	0.6	2.0	0.4
9	Bicycloelemene	1330	t						t	t			t	t
18	Eugenol	1327	0.1	t	t	0.1	0.1	0.4	t	0.1	t	0.1	t	0.1
38	Thymol acetate	1327		t		t					t	0.1		
82	δ-Elemene	1332	0.1	t	t	t	t	t	0.1	0.1	t	t	0.1	0.1

#	Components	RI	OV2 _S	OV2 _C	OV3 _S	OV3 _C	OV16 _S	OV16 _C	OV20 _S	OV20 _C	OV21 _S	OV21 _C	OV23 _S	OV23 _C
55	α-Cubebene	1345	t		t		t	t	t	t	t	t	t	t
65	α-Ylangene	1371	t		t				t	t	t	t	t	t
54	α-Copaene	1375	t	t	t	t	t	0.1	0.1	t	t	t	t	0.1
67	β-Bourbonene	1379	0.1	t	t	t	t	0.1	0.1	0.1	t	t	0.1	0.1
72	β-Elemene	1388	t		t	t			0.1	t			t	0.1
68	β-Caryophyllene	1414	1.8	2.4	1.5	0.2	1.4	0.9	2.0	2.3	1.2	0.8	2.0	1.7
70	β-Copaene	1426	t	t	t		t	t	t	0.1	t	t	t	t
7	Aromadendrene	1428	0.2	0.3	0.3	t	0.2	0.1	0.1	0.1	0.2	0.2	t	t
58	α-Humulene	1447	0.3	0.2	0.2	t	0.2	0.2	0.4	0.4	0.2	0.1	0.4	0.3
5	*allo*-Aromadendrene	1456	t	t	t	t	t	t	t	t	t	t	t	t
78	γ-Muurolene	1469	0.1	0.2	0.1	t	0.2	0.1	t	0.1	0.1	0.1	t	t
20	Germacrene D	1474	0.9	0.6	0.4	t	0.5	0.5	1.4	1.6	0.3	0.2	1.1	2.0
76	β-Selinene	1476	t	t	t						t	t		
50	Valencene	1484	t	t	t				t	t	t	t		
10	Bicyclogermacrene	1487	1.2	0.6	0.7	t	0.5	0.3	1.5	1.0	0.6	0.4	0.7	0.9
59	α-Muurolene	1494	t	t	t	t	t	t	t	t	t	t	t	t
39	*trans,trans*-α-Farnesene	1500	0.3	0.3	0.2		0.2	0.4	0.3	0.3	0.1	0.1	0.3	0.3
66	β-Bisabolene	1500	0.3	0.3	0.2	0.1	0.2	0.4	0.3	0.3	0.1	0.1	0.3	0.3
77	γ-Cadinene	1500	0.3	0.3	0.2	0.1	0.2	0.4	0.3	0.3	0.1	0.1	0.3	0.3
81	δ-Cadinene	1505	0.2	0.4	0.3	t	0.3	0.3	0.1	0.2	0.2	0.2	0.1	0.1
52	α-Cadinene	1529	t	t	t		t	t	t	t	t	t	t	t
46	*trans*-α-Bisabolene	1536	0.1	t	0.2		t	t	t	t	t	t	t	t
33	Spathulenol	1551	0.3	0.2	0.2	t	0.2	0.2	0.3	0.3	0.4	0.1	0.2	0.2
69	β-Caryophyllene oxide	1561	0.1	0.1	t		0.1	t	0.1	0.2	0.2	t	0.2	0.1
21	Globulol	1566	t	t	t		t		t	t	t	t	t	t
51	Viridiflorol	1569	t	t					t	t	t	t	t	t
53	α-Cadinol	1630	t		t		t	t	0.1	t	t	t	t	t
56	α-Eudesmol	1634	0.1		t		0.1	t	0.2	t	0.1	t	t	t
	**% Identification**		100.0	99.9	100.0	100.0	99.6	99.7	99.9	100.0	99.8	100.0	100.0	99.9
	**Grouped Components**													
	Monoterpene hydrocarbons		15.8	12.4	21.6	2.4	10.6	9.6	3.3	8.6	22.4	11.1	5.0	3.0
	Oxygen-containing monoterpenes		77.4	80.9	73.5	96.3	84	85.4	87.6	82.7	71.7	86.1	87.8	88.5
	Sesquiterpene hydrocarbons		5.9	5.6	4.3	0.4	3.9	3.8	6.8	6.9	3.1	2.3	5.4	6.3
	Oxygen-containing sesquiterpenes		0.5	0.3	0.2	0	0.4	0.2	0.7	0.5	0.7	0.1	0.4	0.3
	Phenylpropanoids		0.1	t	t	0.1	0.1	0.4	t	0.1	t	0.1	t	0.1
	Others		0.5	0.7	0.4	0.8	0.6	0.3	1.5	1.2	1.9	0.5	1.6	1.7

#: Number used in PCA analysis. RI: Laboratory-calculated retention index for C_9 to C_{17} *n*-alkanes on DB-1 column. t: traces (<0.05%).

Table S3. Percentage composition of volatile compounds obtained by HS-SPME from *Origanum vulgare* subsp. *virens* accessions from Estremoz, Elvas, Sousel, Serpa, Alandroal and Moura. For samples codes, see Table 1.

#	Components	RI	OV2_S	OV2_C	OV3_S	OV3_C	OV16_S	OV16_C	OV20_S	OV20_C	OV21_S	OV21_C	OV23_S	OV23_C
64	α-Thujene	924	0.8	0.6	0.8	0.3	1.1	0.5	0.1	0.4	0.7	1.0	0.4	t
61	α-Pinene	930	0.3	0.3	0.4	t	0.5	t	t	t	0.2	0.4	t	t
32	Sabinene	958	0.6	0.3	0.3	0.2	0.6	0.3	0.4	0.5	0.4	0.4	0.4	0.5
75	β-Pinene	963	0.6	0.3	0.3	0.2	0.6	0.3	0.4	0.5	0.4	0.4	0.4	0.4
73	β-Myrcene	975	2.1	1.1	1.6	0.8	2.3	0.5	2.1	1.8	0.9	1.6	3.1	1.7
60	α-Phellandrene	995	0.2	t	0.1	t	0.2	0.6	0.9	0.6	0.8	0.2	1.1	0.7
80	δ-3-Carene	1000	0.2	0.1	0.1	0.1	0.2	0.2	0.2	0.1	t	t	0.2	
62	α-Terpinene	1002	1.3	1.1	1.3	0.6	2.4	0.8	0.1	0.5	0.7	1.7	0.4	t
31	*p*-Cymene	1003	2.9	2.7	3.2	1.9	4.1	1.8	0.7	2.1	2.7	3.5	1.3	0.7
23	Limonene	1009	0.1	0.1	0.2	t	0.5		0.2	t		0.2	0.2	
17	*cis*-β-Ocimene	1017	1.7	0.7	1.4	0.3	1.2	0.9	1.6	1.7	2.0	1.2	1.7	1.1
48	*trans*-β-Ocimene	1027	0.3	0.1	0.2	t	0.3		0.6	0.5	0.3	0.2	0.5	0.5
79	γ-Terpinene	1035	13.6	9.9	14.6	6.3	19.3	6.5	1.4	4.2	14.2	12.1	2.1	0.9
2	2,5-Dimethyl styrene	1045			t				0.3	0.3		t	0.4	0.4
35	Terpinolene	1064	0.1	t	t	t	t		t	t		0.1	t	
16	*cis*-Sabinene hydrate	1066	0.3	0.2	0.2	0.1	0.3	1.4	0.5	t	0.2	0.1	t	t
24	Linalool	1074	13.2	2.3	1.1	1.6	6.8	2.1	39.2	24.0	2.8	1.1	46.4	34.7
6	*allo*-Ocimene	1100	0.3	0.1	0.2	t	0.2	t	0.4	0.3	0.3	0.2	0.7	0.2
11	Borneol	1134	0.3	0.1	0.2	0.1	0.2	t	0.3	0.2	0.3	0.1	0.3	0.1
34	Terpinen-4-ol	1148	0.3	0.3	0.3	0.2	0.3	0.3	0.2	0.3	0.3	0.2	0.2	0.1
63	α-Terpineol	1159	0.2	0.1	0.2	0.1	0.9	t	0.2	0.1	0.2	0.1	0.1	t
28	Methyl thymol	1210	1.5	1.2	1.5	1.7	0.6	1.6	0.7	2.2	4.6	1.1	0.8	1.2
25	Methyl carvacrol	1224	3.2	1.6	3.6	3.4	2.1	0.7	1.2	1.7	3.3	3.3	2.0	1.3
37	Thymol	1275	26.4	28.0	10.7	5.5	34.7	47.9	17.6	30.3	36.8	54.8	9.8	21.8
13	Carvacrol	1286	11.3	32.8	41.8	66.7	5.1	17.5	9.1	4.1	8.7	0.8	8.2	11.2
18	Eugenol	1327	0.2	0.3	0.2	0.2	0.1	0.3	t	0.1		0.2	t	0.1
82	δ-Elemene	1332	1.0	0.8	0.6	0.6	0.3	1.8	1.5	1.3	1.0	0.6	1.2	1.2
55	α-Cubebene	1345	t	0.1	0.1	t	0.1	t	1.4	1.4	t		t	t
65	α-Ylangene	1371	t				0.1		t	t	t	t		
54	α-Copaene	1375	0.1	0.2	0.2	t	0.2	t	t	t	0.1	0.2	t	t
67	β-Bourbonene	1379	0.1	0.1	0.1	t	0.1	0.2	0.3	0.4	0.1	0.1	0.2	0.4
71	β-Cubebene	1385		t	t	t	0.1		0.1	t		t	t	t
68	β-Caryophyllene	1414	5.1	5.0	4.0	2.3	4.7	2.5	5.9	5.6	5.0	3.4	5.5	5.6
70	β-Copaene	1426	0.2	0.3	0.2	0.1	0.2	0.1	0.2	0.3	0.3	0.2	0.3	0.3
7	Aromadendrene	1428	0.9	0.9	1.0	0.5	0.7	0.7	0.5	0.8	1.1	1.0	0.4	0.9
58	α-Humulene	1447	0.8	0.6	0.5	0.2	0.6	0.4	1.0	1.0	0.7	0.6	0.9	0.9
47	*trans*-β-Ionone	1452	0.2	0.2	0.2	0.3	0.1	0.4	0.3	0.5	0.3	0.2	0.2	0.5
78	γ-Muurolene	1469	0.4	0.5	0.5	0.3	0.5	0.3	0.2	0.4	0.5	0.5	0.2	0.3
20	Germacrene D	1474	2.0	0.9	1.1	0.7	1.2	1.3	3.6	3.6	1.3	1.5	2.7	4.2
10	Bicyclogermacrene	1487	1.6	0.8	1.1	0.4	0.9	0.4	2.3	1.1	1.5	1.0	1.0	1.6
59	α-Muurolene	1494	0.1	0.1	0.1	t	0.2	t		0.2	0.2	0.2	t	t
39	*trans,trans*-α-Farnesene	1500	0.1	0.1	0.1	0.1	0.1	0.3	0.1	0.1	0.1	0.1	0.1	0.1
66	β-Bisabolene	1500	2.6	2.4	2.1	2.5	2.0	4.2	3.2	3.9	2.0	1.9	2.4	3.9
41	*trans*-Calamenene	1505	t	t						t	t		t	t
81	δ-Cadinene	1505	0.6	0.7	0.6	0.4	0.6	0.5	0.4	0.5	0.7	0.7	0.3	0.5
33	Spathulenol	1551	0.2	t	0.4	0.2	0.2	t	0.1	0.6	0.3	0.3	0.2	t
69	β-Caryophyllene oxide	1561	t	t	0.2	0.1	t	t	t	0.3	t	0.1	0.1	t

#	Components	RI	OV2_S	OV2_C	OV3_S	OV3_C	OV16_S	OV16_C	OV20_S	OV20_C	OV21_S	OV21_C	OV23_S	OV23_C
26	Methyl dihydrojasmonate	1616		t	t	t	t	0.2					t	t
	**% Identification**		98.0	98.0	97.6	99.0	97.5	97.5	99.5	98.5	96.0	97.6	96.4	98.0
	**Grouped Components**													
	Monoterpene hydrocarbons		25.1	17.4	24.7	10.7	33.5	12.4	9.1	13.2	23.6	23.2	12.5	6.7
	Oxygen-containing monoterpenes		56.7	66.6	59.6	79.4	51.0	71.5	69.3	63.2	57.2	61.6	68.2	70.8
	Sesquiterpene hydrocarbons		15.6	13.5	12.3	8.1	12.6	12.7	20.7	20.6	14.6	12.0	15.2	19.9
	Oxygen-containing sesquiterpenes		0.4	0.2	0.8	0.6	0.3	0.4	0.4	1.4	0.6	0.6	0.5	0.5
	Phenylpropanoids		0.2	0.3	0.2	0.2	0.1	0.3	t	0.1	t	0.2	t	0.1
	Others		t	t	t	t	t	0.2	t	t	t	t	t	t

#: Number used in PCA analysis. RI: Laboratory-calculated retention index for *n*-alkanes from C_9 to C_{17} on the DB-1 column. t: trace (< 0.05%).

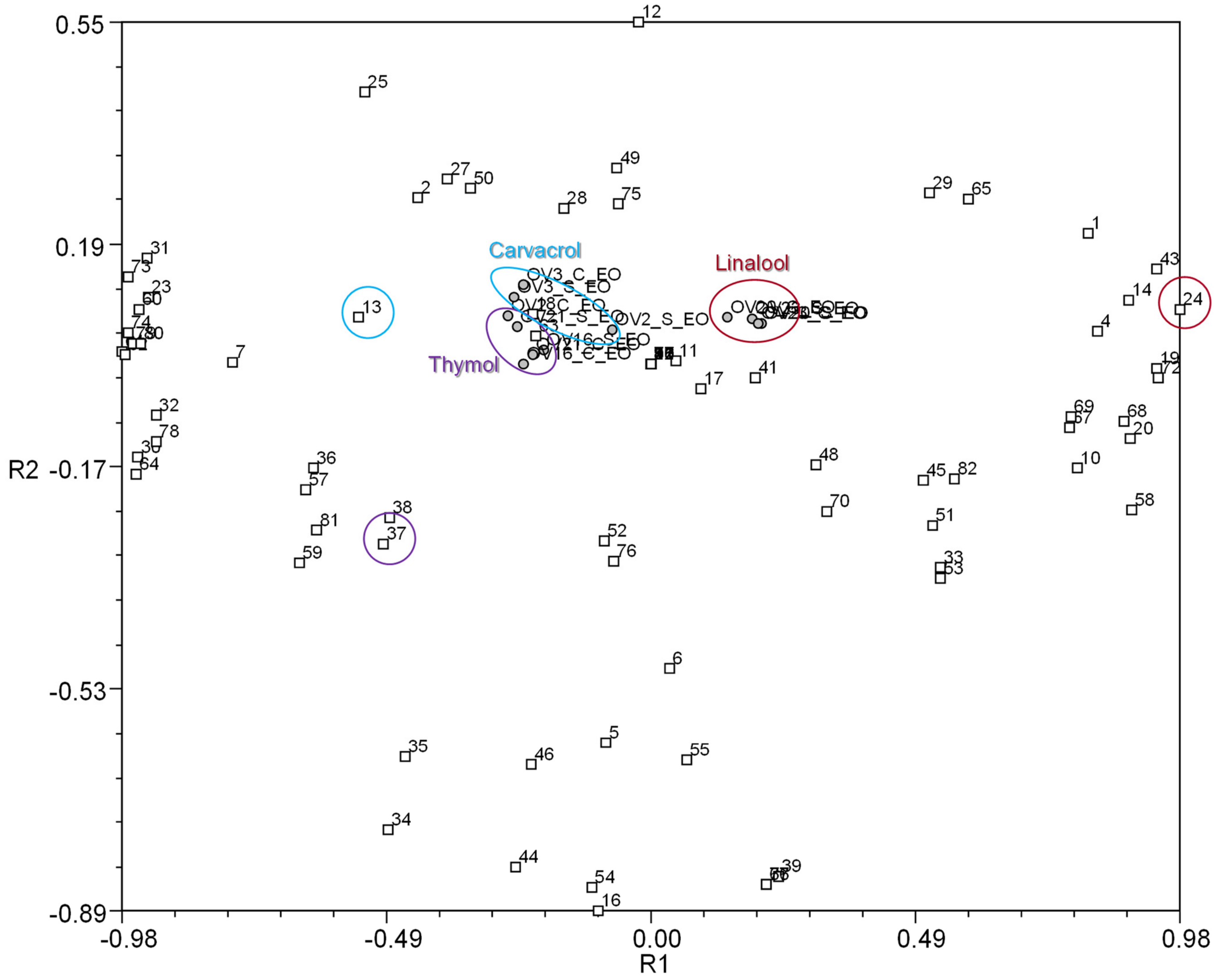

Figure S1. Graph obtained by PCA analysis of the percentage composition of the essential oils (EO), isolated from 12 *Origanum vulgare* subsp. *virens* samples, based on correlation. Main compounds / chemotypes: carvacrol (number label 13, light blue), thymol (number label 37, purple) and linalool (number label 24, dark red). For other compounds number label, see Tables 2–5 and S1–S3. For samples codes, see Table 1.

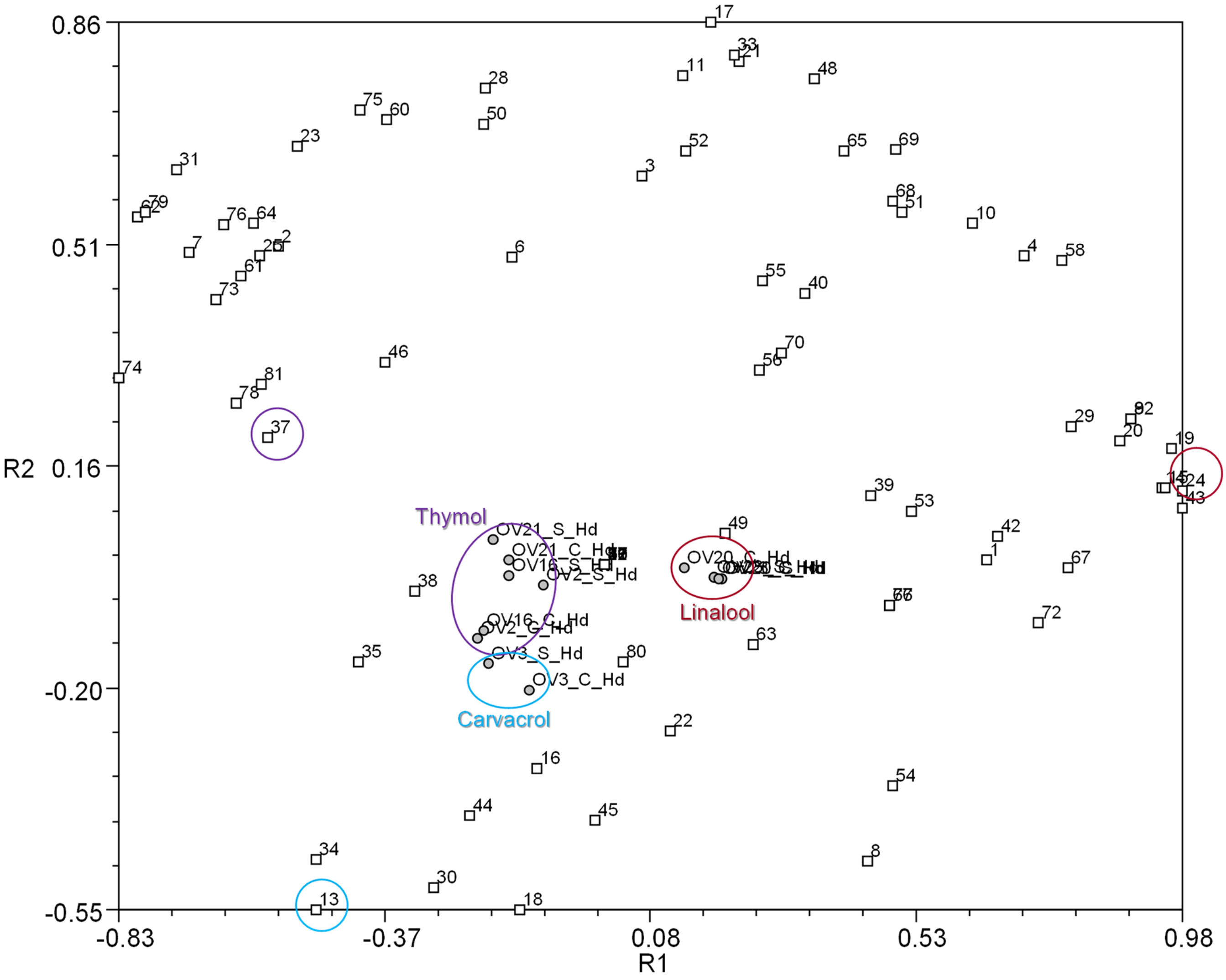

Figure S2. Graph obtained by PCA analysis of the percentage composition of the hydrolate volatiles (Hd), isolated from 12 *Origanum vulgare* subsp. *virens* samples, based on correlation. Main compounds / chemotypes: carvacrol (13, light blue), thymol (37, purple) and linalool (24, dark red). For other compounds number label, see Tables 2–5 and S1–S3. For samples codes, see Table 1.

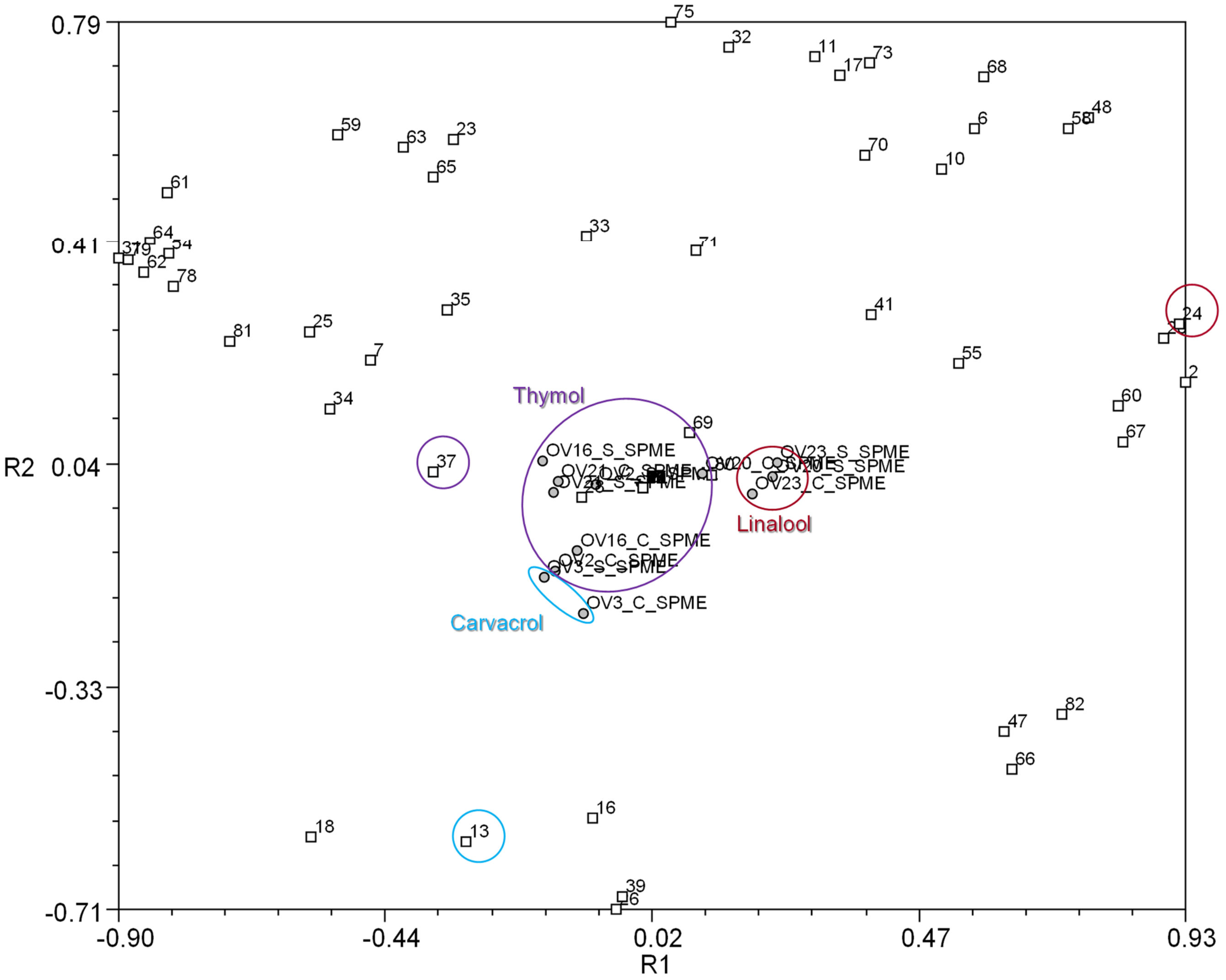

Figure S3. Graph obtained by PCA analysis of the percentage composition of HS-SPME volatiles (SPME), isolated from 12 *Origanum vulgare* subsp. *virens* samples, based on correlation. Main compounds / chemotypes: carvacrol (13, light blue), thymol (37, purple) and linalool (24, dark red). For other compounds number label, see Tables 2–5 and S1–S3. For samples codes, see Table 1.
